# Functional Recovery Following Pertrochanteric Hip Fractures Fixated with the Dynamic Hip Screw vs. the Percutaneous Compression Plate

**DOI:** 10.1100/tsw.2005.29

**Published:** 2005-03-19

**Authors:** Yocheved Laufer, Miriam Lahav, Reuben Lenger, Elliot Sprecher

**Affiliations:** ^1^ Department of Physical Therapy, University of Haifa, IL-31905 Haifa, Israel; ^2^ Physical Therapy Department, Clalit Health Services, Tivon, Israel; ^3^ Flieman Geriatric Rehabilitation Center, Haifa, Israel; ^4^ Department of Neurology, Rambam Medical Center, Haifa, Israel

**Keywords:** pertrochanteric hip fracture, fixation, dynamic hip screw, percutaneous compression plate, functional recovery, rehabilitation, weight bearing, pain, activities of daily living, assistive devices, Israel

## Abstract

The Dynamic Hip Screw (DHS) is currently the most frequently used implant for the treatment of pertrochanteric hip fractures. The Percutaneous Compression Plate (PCCP) is a recently developed, alternative device that involves minimal invasive surgery. The objective of the present study was to compare functional recovery following these two surgical procedures. A total of 76 consecutive elderly subjects (mean age and standard deviation, 80.6 ± 5.5) following pertrochanteric hip fracture fixation were evaluated prospectively. Functional recovery was assessed 3 and 12 weeks and 2 years following surgery. Differences between groups 3 weeks postsurgery were found only in pain level during ambulation and in the weight-bearing capability of the operated extremity, which were both in favor of the PCCP. By 3 months, both groups had improved in all measures, but did not reach their preinjury level of independence. However, the PCCP group ambulated with fewer assistive devices and demonstrated better recovery of basic activities of daily living (BADL). While the majority of the subjects from both groups ambulated independently 2 years postsurgery, the PCCP group exhibited less pain during ambulation, was more independent in ADL, and required fewer assistive devices for ambulation. To summarize, the PCCP presents enhanced short- and long-term recovery of functional abilities in comparison to DHS. However, given the limited number of patients, further studies are necessary to substantiate these results.

## INTRODUCTION

High incidence of hip fractures among the elderly is a worldwide major health problem with severe medical and socioeconomic consequences affecting mortality and quality of life among the aging population[1,2,3]. There is broad consensus in the medical profession that the return to prefracture functional independence is the primary treatment goal following a hip fracture and that in most cases, surgical management with early mobilization is the treatment of choice for achieving this goal[1,2,4,5].

The Dynamic Hip Screw (DHS) is the device most commonly used for the internal fixation of pertrochanteric hip fractures[4,6] and involves a closed reduction with open internal fixation[4]. Depending on the stability of the fracture, weight bearing as tolerated is usually recommended for the period of 4–6 weeks following surgery[2,7]. Although the majority of patients heal well, certain medical and orthopedic complications prevail[8,9,10] and many of the patients do not regain their prefracture level of ambulation and independence in activities of daily living (ADL) 1 year following surgery[1,3,11,12].

The Percutaneous Compression Plating (PCCP) is a relatively new procedure for pertrochanteric hip fracture fixation that offers minimal operative trauma[13,14]. The device consists of a plate with three cortical shaft screws and two dynamic neck screws, which are inserted percutaneously by way of two 2-cm incisions and assembled within the patient. It is postulated that the double dynamic screws, as opposed to the single screw used in the DHS, offer additional fracture stability by reducing possible rotational torque[15]. Weight bearing as tolerated is encouraged immediately postsurgery. Comparative studies are inconclusive regarding the advantages of the PCCP vs. the DHS in terms of bone healing time, fracture stability, and complications[16,17,18]. However, the PCCP has been shown to provide adequate bending stiffness and torsional stability[15], as well as to require shorter surgical time and less frequent blood transfusions[16,17,18]. Furthermore, patients following PCCP reported less pain during the first postoperative week[16], which is probably related to the sparing of bone and soft tissue as well as to the presence of fewer hematomas[17].

Unlike the DHS, which has been extensively studied[4,6], the PCCP has received limited research attention. In an attempt to bridge this gap, the present study was designed to compare the effect of these two surgical approaches on the functional rehabilitation outcome of elderly subjects following a pertrochanteric hip fracture.

## METHODS

All medically stable patients following surgery for hip fracture in three acute-care hospitals in Israel were referred to a geriatric rehabilitation center and screened during a 9-month period of data collection. Two of the referring hospitals routinely performed the DHS procedure and one hospital the PCCP procedure. Therefore, the type of surgery that the patients underwent depended on the general hospital to which they had been assigned post-trauma. Inclusion criteria were as follows:

1. Presentation of a pertrochanteric hip fracture classified from the preoperation Roentgen films as either stable (31-A1 and 31-A2.1) or unstable (31-A2.2 and 31-A2.3) in accordance with the classification of the Orthopaedic Trauma Association[19]
2. Minimal age of 65 years
3. Ability to ambulate independently prior to the fracture with or without assistive devices
4. Up to 3 weeks between surgery and admittance to the rehabilitation center
5. No cognitive or other medical condition that could hamper ability to participate in the study

The study was approved by the Institutional Review Board and all subjects signed informed consent forms prior to participation.

The subjects were evaluated three times. The first evaluation (T1) was conducted between 2–3 weeks postsurgery at the rehabilitation center and included the following components:

1. The Functional Recovery Score (FRS) regarding functional status **prior** to the current fracture. The FRS is a questionnaire designed to assess the restoration of function of elderly post–hip fracture patients and its reliability and validity have been previously demonstrated[20,21]. The total FRS score of 100% was comprised of three main parts:

a. Basic activities of daily living (BADL) as assessed by four items and assigned 44% of the total score
   b. Instrumental activities of daily living (IADL) as assessed by six items and assigned 23% of the total score
   c. Mobility as assessed by one item and assigned 33% of the total score

Each item is scored between 0–4[20,21].

2. Percentage of body weight (%BW) borne by the involved and uninvolved lower extremities (LE) was measured via computerized force plates (The Tetrax Portable Multiple System, 56 Miryam ST., Ramat Gan, Israel). Weight bearing was measured during 30 sec of quiet stance in the following three test conditions:
   a. In a relaxed symmetrical weight-bearing stance
   b. While shifting maximal weight to the uninvolved extremity
   c. While shifting maximal weight to the operated extremity

All tests were conducted without the use of an assistive device and test order was randomized.
3. Pain evaluation during ambulation and at night using a 10-point Visual Analogue Scale (VAS)[22]
4. Assistive device used for ambulation.

A 3-month follow-up examination (T2) was carried out at the permanent residence of the subjects. Only subjects who were available for both T1 and T2 were included in the study. Evaluation at T2 differed from T1 in the following: (a) the FRS referred to the functional abilities of the subjects at the time of the evaluation and (b) weight-bearing pattern was not evaluated. Finally, a 2-year follow-up evaluation (T3) was conducted via a phone interview when the patients and/or the patient’s primary caretaker answered the FRS questionnaire and reported use of assistive devices for ambulation. The researcher conducting all evaluations was blind to the type of surgery of the subjects at T1 and T2.

Comparisons between groups for pain and FRS variables at T1 and T2 employed repeated-measures ANOVA, with group and time as the repeated factors and the interaction of group and time as the independent variables. In order to improve the normality of data distributions of the FRS variables, scores of each part were converted to percentages, to which an arcsine square root transformation was applied. The analyses of the weight distributions employed repeated-measures ANOVA based on data at T1, with group and stance as the repeated factors, and the interaction of group and stance as the independent variables. Adjusted Tukey-Kramer tests were utilized to compare predetermined individual conditions. For the purpose of analysis, devices were divided into two groups (devices 1–7 and devices 8–9, see Table 4). Since use of device at T1 was determined by the hospitals’ routine and preference of the treating physical therapist, analysis comparing utilization of devices was determined only at T2 and T3 using a two-tailed Fisher Exact test applied at each evaluation. The follow-up comparisons at T3 involved much smaller groups and were thus treated separately so as not to degrade the analyses comparing T1 and T2. T-tests were utilized in this evaluation, with conversion to percentage and arcsine square root transformation again applied to the FRS data.

## RESULTS

Of the 76 subjects who met the inclusion criteria, 17 were lost at the 3-month follow-up and excluded from the study. Characteristics of the remaining 59 subjects (29 following DHS and 30 following PCCP) and causes for exclusion by type of surgery are presented in Table 1. There were no significant differences between groups in terms of age, gender, time between surgery and evaluations, use of assistive devices prior

**TABLE 1**
**Characteristics of Included Patients Examined at T1 and T2, and Causes for Unavailability at T2 Leading to Exclusion from Study**

| Patients Tested at T1 and T2 | DHS (n = 29) | PCCP (n = 30) |
|---|---|---|
| Number (male/female) | 29 (7/22) | 30 (10/20) |
| Age (years) | 81.1 ± 5.3 | 80.0 ± 6.0 |
| Assistive devices prior to fall (none/use) | 19/10 | 22/8 |
| Days between surgery and T1 | 18.8 ± 5.1 | 16.9 ± 5.7 |
| Days between surgery and T2 | 90.2 ± 23.8 | 98.4 ± 18.8 |
| Fracture side (left /right) | 14/15 | 16/14 |
| Stable/unstable | 11/18 | 13/17 |
| Residence prior to fracture: | | |
| Home | 28 | 30 |
| Nursing home | 1 | 0 |
| Residence postfracture: | | |
| Home | 18 | 21 |
| Family | 8 | 7 |
| Nursing home | 3 | 2 |
| **Causes for Exclusion from Study (Unavailable at T2)** | | |
| Refusal | 3 | 2 |
| Full nursing care | 3 | 4 |
| Medical complication | 1 | 2 |
| Fracture of lateral wall | 2* | 0 |

* Subjects diagnosed with a fracture of the lateral wall within 3 months from original fracture requiring surgical revision.

to surgery, type of residence prior to fracture, side of fracture, and fracture stability. Characteristics of the 44 patients who participated in the 2-year follow-up and causes for drop out are presented in Table 2. Results of outcome measures are presented in Tables 3 and 4.

ANOVA employed to compare surgery groups at T1 and T2 indicated that overall FRS was significantly lower at 3 months postfracture than at prefracture ($p = 0.0001$). Although ANOVA indicated a significant difference in total FRS between the surgery groups ($p = 0.021$), as no interaction effect was observed between groups and test time, this difference is most probably due to the marginal difference between groups at the prefracture examination ($p = 0.073$). Separate analyses of the IADL and mobility scores indicated significantly lower scores at 3 months postfracture ($p = 0.0001$) and no effect of surgery or interaction between time and surgery. Only the BADL variable demonstrated a different pattern. Although neither surgery group recovered their BADL ability to prefracture level ($p = 0.0001$), a strong interaction effect between time and surgery ($p = 0.0089$) indicated that recovery of BADL of the PCCP group was significantly greater than that of the DHS group.

ANOVA indicated a significant effect of stance condition on weight distribution with no difference between the patient groups and no interaction effect between group and stance condition. Post hoc tests indicated no difference between groups when subjects were required to maintain a relaxed symmetrical stance (mean %BW ± SD on the involved LE for DHS and PCCP is 39.50 ± 12.74 and 41.39 ± 9.68, respectively) or when required to shift maximum weight to the uninvolved LE (mean %BW ± SD on the uninvolved LE for DHS and PCCP is 66.4 ± 14.0 and 66.49 ± 15.3, respectively). However, when patients were required to place maximally tolerated weight on the involved side, there was a significant increase to the involved side only in the PCCP group ($p = 0.006$) (mean %BW ± SD on the involved LE for DHS and PCCP is 43.12 ± 12.32 and 49.32 ± 11.75, respectively).

**TABLE 2**
**Subject Characteristics of Patients Included in the 2-Year Follow-Up and Causes for Unavailability for Follow-Up**

| Patients at 2-Year Follow-Up | DHS (n = 22) | PCCP (n = 22) |
|---|---|---|
| Number (male/female) | 6/16 | 6/16 |
| Age (years) | 84.5 ± 5.8 | 82.4 ± 5.3 |
| Time since surgery (years) | 2.49 ± 0.15 | 2.44 ± 0.26 |
| **Causes for Drop Out from 2-Year Follow-Up** | | |
| Death | 6 | 3 |
| Surgical revision* | — | 1 |
| Fracture other extremity** | 1 | 2 |
| Alzheimer | — | 1 |
| Blindness | 1 | — |

* Subject fell due to dizziness and refractured the femur.
** Due to fall.

**TABLE 3**
**Mean ± SD FRS, Pain Level, and Walking Speed of Subjects at the First (T1), Second (T2), and Third (T3) Evaluations**

| FRS | DHS | | | PCCP | | |
|---|---|---|---|---|---|---|
| | T1 | T2 | T3 | T1 | T2 | T3 |
| BADL (0–4) | 3.77 ± 0.4 | 2.68 ± 0.9† | 2.96 ± 1.1‡ | 3.75 ± 0.49 | 3.22 ± 0.75† | 3.65 ± 0.5‡ |
| IADL (0–4) | 2.20 ± 1.3 | 0.61 ± 0. 9 | 0.83 ± 1.2‡ | 2.70 ± 1.47 | 1.01 ± 1.12 | 1.9 ± 1.6‡ |
| Mobility (0–4) | 3.47 ± 0.5 | 2.17 ± 0.9 | 2.36 ± 1.1‡ | 3.90 ± 1.67 | 2.64 ± 0.83 | 2.95 ± 0.8‡ |
| Total (%) | 82.96 ± 14.6 | 49.2 ± 17.4 | 57.1 ± 25.2‡ | 87.2 ± 17.4 | 62.8 ± 20.6 | 75.4 ± 18.2‡ |
| **Pain (VAS)** | | | | | | |
| At night | 4.51 ± 3.3 | 2.14 ± 3.2 | 0.8 ± 1.8 | 2.83 ± 3.2 | 1.33 ± 2.2 | 0.09 ± 0.4 |
| While walking | 5.82 ± 3.0* | 2.93 ± 3.2 | 2.27 ± 2.8‡ | 3.96 ± 2.7* | 2.70 ± 2.3 | 0.77 ± 1.5‡ |

*Note*: BADL, basic activities of daily living; IADL, instrumental activities of daily living; VAS, visual analogue scale.
***** Significant difference at T1.
† Significant difference at T2.
‡ Significant difference at T3.

ANOVA indicated a significant reduction in night pain by T2 ($p = 0.0001$) with no difference between groups and no interaction effect between group and test time. The results also showed a significant reduction in walking pain by T2 ($p = 0.0001$), though walking pain was affected by type of surgery as well ($p = 0.05$). This difference between groups was present only at T1, when the pain reported by the PCCP group was significantly lower ($p = 0.05$), resulting in a marginal interaction effect between test time and group ($p = 0.08$). At T2, more patients in the PCCP ambulated with less external support (a single cane or no cane) than patients in the DHS group ($p = 0.029$).

**TABLE 4**
**Assistive Devices Used by Subjects at the First (T1), Second (T2), and Third (T3) Evaluations**

| | | | DHS | | | PCCP | | |
|---|---|---|---|---|---|---|---|---|
| | | | T1 | T2 | T3 | T1 | T2 | T3 |
| Group | | Device | Number (%) | | | | | |
| One | 1 | Nonambulatory | — | — | 2 (9) | — | — | — |
| | 2 | Forearm walker | 5 (17) | — | — | 6 (20) | — | — |
| | 3 | Rolator | 11 (38) | 4 (14) | 2 (9) | 8 (27) | 1 (3) | — |
| | 4 | Walker | 12 (41) | 7 (24) | 6 (27) | 15 (50) | 4 (13) | 4(18) |
| | 5 | 2 Quad-canes | 1 (3) | 6 (21) | — | 1 (3) | 5 (17) | — |
| | 6 | 1 Quad-cane | — | 3 (10) | 2 (9) | — | 2 (7) | — |
| | 7 | 2 Canes | — | 3 (10) | 1 (5) | — | 3 (10) | — |
| Two | 8 | 1 Cane | — | 6 (21) | 8 (36) | — | 12 (40) | 11 (50) |
| | 9 | No device | — | — | 1 (5) | — | 3 (10) | 7 (32) |

T-test analysis of the FRS at the 2-year follow-up indicated that the PCCP group demonstrated higher overall FRS scores ($p$ = 0.009), BADL scores ($p$ = 0.0213), IADL scores ($p$ = 0.02), and mobility scores ($p$ = 0.046) than the DHS group. Thus, at T3 the PCCP group obtained 97, 70, and 76% of their score at T1 for BADL, IADL, and mobility, respectively. In contrast, the DHS group obtained at T3 only 79, 38, and 68% of their T1 scores for the same categories. While no differences between groups were observed in regard to night pain, the PCCP group complained of less pain during ambulation ($p$ = 0.033) and used less supportive devices than the DHS group ($p$ = 0.012).

## DISCUSSION

The primary goal of surgical treatment following hip fracture is the patient's return to the prefracture level of functional independence. The 3-month follow-up results provide support for previous studies indicating that although the functional ability of elderly patients is dramatically enhanced 3 months postsurgery in comparison with the immediate postsurgery period, the majority of patients do not reach their prefracture level of independence at the 3-month milestone[21].

It is generally accepted that early postoperative ambulation is vital for the prevention of medical complications and that restricted weight bearing can delay functional recovery of the elderly patient[23]. Furthermore, it is postulated that provided fracture stability is maintained, fracture impaction achieved by cyclic loading during the weight-bearing phase of locomotion, accompanied by fixation devices with sliding capabilities, will enhance bone healing[14]. Regardless of whether postoperative instructions are full or partial weight bearing, early ambulation involves the use of assistive devices with patients seeming to voluntarily limit loading on the injured limb[24]. However, very little information is available concerning the actual weight bearing assumed by patients postsurgery. In this study, weight bearing as tolerated was recommended to all patients. Yet, neither group was able to bear on the fractured LE the 66% of body weight that they could shift to the uninvolved LE. When subjects were requested to shift from a relaxed stance and to place maximum weight on the operated LE, the percentage of body weight on the fractured limb changed significantly only in patients following PCCP. These results may indicate a higher level of comfort with weight bearing in the PCCP group, which may be associated in the early stages of recovery with the reduced invasiveness of the PCCP procedure. In turn, this may explain the greater reduction in postoperative pain during ambulation also previously reported[16] and the reduction in the use of assistive devices observed in this group. However, these results should be regarded with caution as weight distribution was examined only during static stance, which may not truly simulate the more dynamic pattern of ambulation.

The use of less external support at the 3-month follow-up was not associated with differences in overall FRS, IADL, or mobility. The only functional difference between groups, which favored the PCCP group, was the ability to perform basic home activities (BADL). The greater difficulty in recovering instrumental activities observed in both groups has been previously noted[12,25]. These results suggest that factors other than independent ambulation in the safe home environment determine the ability of patients to implement this skill outdoors and to achieve the ultimate goal of rehabilitation involving participation in community life.

Given the relatively recent development of the PCCP implant, no long-term follow-up studies have yet been reported concerning functional rehabilitation following this procedure. Thus, the most interesting results of the present study pertain to the 2-year follow-up. Whereas at the 3-month milestone results indicated a comparable functional recovery between groups with the PCCP group showing enhanced recovery only in BADL and in the use of external support, at the 2-year milestone, the patients with the PCCP implant demonstrated a higher level of functional recovery in all the evaluated parameters. In addition, they continued to utilize less external support for ambulation and reported less pain during ambulation.

The minimally invasive PCCP surgery has been shown to involve less blood loss, shorter operation time, and faster soft tissue healing[17], all of which may have a direct effect on short-term functional recovery, particularly among the elderly population. But these factors seem an unlikely explanation for the long-term enhanced functional ability of the PCCP group. Independence in ADL prior to the fracture has been shown to be an important predictive factor of the ability of patients to regain their prefracture level of independence 1-year postfracture[12]. While the prefracture total FRS score of the PCCP group tended to be higher than the prefracture score of the DHS group (mean score difference of 4%), this too does not provide a likely explanation for the significant 18% difference in functional recovery observed at the 2-year follow-up.

It has been demonstrated that a key to fracture stability and event-free fracture healing lies in the preservation of the lateral wall[9]. It is claimed that the two-screw PCCP device may offer greater rotational stability[15] and that the small diameter of the holes at the drilling site used with the PCCP[13] may be responsible for the preservation of the lateral wall and the reduction in fracture collapse. In the present study, two patients following the DHS procedure were excluded due to a lateral wall fracture, while none of the patients following PCCP demonstrated a similar complication. However, the small number of involved patients does not allow for definitive conclusions regarding the use of one procedure over the other for the prevention of this complication. Further follow-up studies, which will include radiographic data, are necessary to determine whether the observed enhanced recovery of functional capabilities observed in the PCCP group is related to fracture healing. Furthermore, as this preliminary study involved a rather small number of participants, more extensive studies should be conducted to substantiate the results.

## BIOSKETCHES

**Yocheved Laufer, PT, DSc,** is a full-time faculty member at the University of Haifa, Israel, currently in the position of Head of the Physical Therapy Department. Her research interests are in the field of neurological and geriatric rehabilitation. E-mail: yocheved@research.haifa.ac.il

**Miriam Lahav, PT, MSc,** is a practicing physical therapist since 1969, with a MSc in physical therapy since 2001. She is the director of an outpatient clinic specializing in orthopedic and geriatric rehabilitation. E-mail: miri@parasol_pc.co.il

**Reuben Lenger, MD,** is a specialist in physical medicine and rehabilitation, currently Head of the Orthopedic Rehabilitation Department at the Flieman Geriatric Rehabilitation Hospital in Haifa, which is affiliated with the Faculty of Medicine at the Technion. E-mail: lenger@netvision.net.il

**Elliot Sprecher, PhD,** is an experimental psychologist and biomedical research consultant affiliated with the Department of Neurology, Rambam Medical Center, Haifa, Israel. E-mail: elliots@netvision.net.il
